# Reliability and Failure Mode of Ti-Base Abutments Supported by Narrow/Wide Implant Systems

**DOI:** 10.3390/dj11090207

**Published:** 2023-08-29

**Authors:** Ernesto B. Benalcázar-Jalkh, Laura F. de Carvalho, Larissa M. M. Alves, Tiago M. B. Campos, Edisa de Oliveira Sousa, Edmara T. P. Bergamo, Paulo G. Coelho, Petra C. Gierthmuehlen, Frank A. Spitznagel, Abbas Zahoui, Estevam A. Bonfante

**Affiliations:** 1 Department of Prosthodontics and Periodontology, Bauru School of Dentistry, University of Sao Paulo, Bauru 17012-980, Brazil; 2 Department of Surgery, University of Miami Miller School of Medicine, Miami, FL 33136, USA; 3 Department of Prosthodontics, Medical Faculty and University Hospital Düsseldorf, Heinrich-Heine University, Moorenstraße 5, 40225 Düsseldorf, Germany

**Keywords:** dental implants, failure mode, fatigue, narrow implants

## Abstract

To assess the reliability and failure modes of Ti-base abutments supported by narrow and wide-diameter implant systems. Narrow (Ø3.5 × 10 mm) and wide (Ø5 × 10 mm) implant systems of two different manufacturers with internal conical connections (16°) and their respective Ti-base abutments (3.5 and 4.5 mm) were evaluated. Ti-base abutments were torqued to the implants, standardized metallic maxillary incisor crowns were cemented, and step stress accelerated life testing of eighteen assemblies per group was performed in three loading profiles: mild, moderate, and aggressive until fracture or suspension. Reliability for missions of 100,000 cycles at 100 and 150 N was calculated, and fractographic analysis was performed. For missions at 100 N for 100,000 cycles, both narrow and wide implant systems exhibited a high probability of survival (≥99%, CI: 94–100%) without significant differences. At 150 N, wide-diameter implants presented higher reliability (≥99%, CI: 99–100%) compared to narrow implants (86%, CI: 61–95%), with no significant differences among manufacturers. Failure mode predominantly involved Ti-base abutment fractures at the abutment platform. Ti-base abutments supported by narrow and wide implant systems presented high reliability for physiologic masticatory forces, whereas for high load-bearing applications, wide-diameter implants presented increased reliability. Failures were confined to abutment fractures.

## 1. Introduction

The rehabilitation of partially and completely edentulous patients through implant-supported prostheses depends on the successful achievement of implant osseointegration. For over five decades, dental implants have been widely used to support single, multiple, and full-arch prostheses, with high survival rates for both implants and implant-supported reconstructions (approximately 95% and 90% after 10 years of follow-up, respectively) [1,2,3,4,5,6,7]. Along with such strong clinical data, the fast-paced development of implant systems and bioengineering have motivated the application of dental implants for the resolution of a variety of challenging clinical scenarios, such as reduced bone quality and availability and immediate implant placement in post-extraction sockets [8,9,10,11].

While implant designs in different length scales have evolved to promote faster and predictable osseointegration in challenging scenarios [12,13,14,15], an optimal three-dimensional positioning of the implant device is crucial to assure success in the long term and to avoid, or at least reduce, biological and mechanical complications [16,17]. Ridge atrophy after tooth extractions, agenesis, trauma, and neoplasia have been reported as challenging scenarios for ideal implant positioning [18]. In clinical scenarios with reduced bone availability, the osteotomy size required for standard platform implant placement may lead to thread exposure, compromising the integrity of the osteotomy walls and the predictability of the treatment. While bone grafting procedures prior to implantation have been suggested as alternatives to preserve the alveolar walls and allow for correct implant positioning, they also promote a significant increase in morbidity, healing time and treatment costs [19,20]. Therefore, the use of implants with a reduced diameter (<3.75 mm) has become a clinical option to rehabilitate areas with limited bone availability [16,17,20,21].

Although there has been more than one attempt to classify dental implant dimensions (length and diameter), Al-Johany et al. have proposed the following four categories regarding diameter: wide (⌀ ≥ 5 mm), standard (⌀ ≥ 3.75 mm and <5 mm), narrow (⌀ ≥ 3.0 mm and <3.75 mm), and extra-narrow (⌀ < 3 mm) implants [22]. Besides the significant differences in bulk structure and clinical indication for different bone availability scenarios, the mechanical evaluation of reconstructions supported by implants with different diameters has shown an increasing probability of survival from narrow to standard and wide implant systems [23]. Under loading, the generated stresses in narrow implants are distributed to a significantly smaller area compared to implants with wide and standard diameters [24]. However, clinical evaluations of narrow systems have reported similar survival rates in comparison to standard diameter implants (>95% after an average of 4 years follow-up and marginal bone loss of ~2 mm) [25,26,27].

Despite the favorable survival rates reported in the clinical literature, the thin implant wall of narrow implants with 16° taper internal conical connection, instead of the more usual 11.5°, may lead to higher risk for fracture due to their thinner structure, particularly considering the damage accumulation when submitted to repetitive physiological loading [28,29]. Considering the detrimental effect of cyclic loads on the mechanical performance of implant systems, fatigue testing has been consistently used in the dental literature to estimate the lifetime and modes of failure of implant-supported reconstructions [23,30,31,32,33]. Therefore, this study aimed to assess the reliability of two narrow implant systems through step stress accelerated life-testing (SSALT) and to compare them with its counterpart, wide implant systems from the same manufacturers, all with a 16° taper internal conical connection. The postulated null hypothesis was that there would be no significant differences in the reliability of narrow and wide implant systems, irrespective of the manufacturer.

## 2. Materials and Methods

### 2.1. Sample Preparation

**Figure 1:**
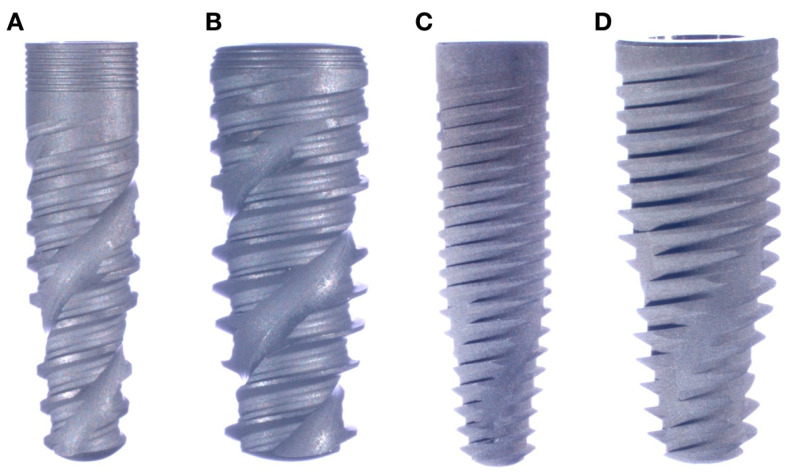
Macrogeometry of tested implants: Epikut 3.5 (**A**), Epikut 5 (**B**), GM 3.5 (**C**), and GM 5 (**D**).

Two implant systems, Epikut (SIN Implants, Sao Paulo, SP, Brazil) and Grand Morse (GM; Neodent, Curitiba, PR, Brazil), with 16° taper internal conical connections, were evaluated in the present study. Eighteen narrow (Ø3.5 × 10 mm) and eighteen wide (Ø5 × 10 mm) implants of each system were rehabilitated with proprietary Ti-base abutments of 3.5 mm and 4.5 mm, respectively, to obtain four experimental groups (n = 18/group): (i) Epikut 3.5, (ii) Epikut 5, (iii) GM 3.5 and (iv) GM 5. Both implant devices are manufactured with cold-worked grade IV titanium and consist of a conical body with a large thread pitch and deep wide threads, as depicted in Figure 1.

**Figure 2:**
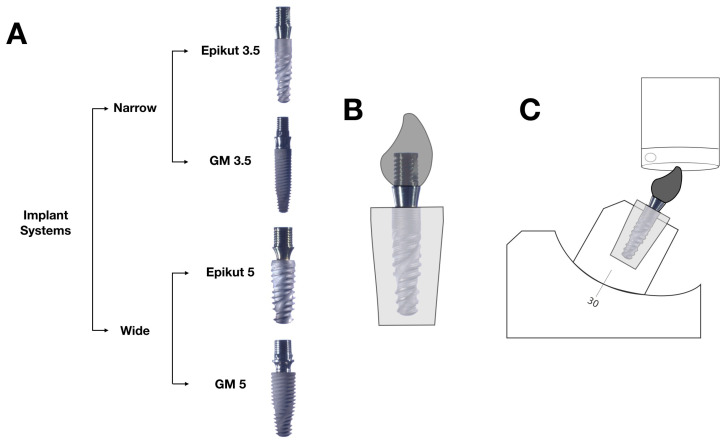
(**A**) Experimental design of the study, where two narrow (3.5 mm) and two wide (5 mm) implant systems (Epikut, SIN, National Implant System, and Grand Morse, Neodent) were tested with their respective proprietary Ti-base abutments. (**B**) Schematic of the implant embedded in acrylic resin up to the platform level with cobalt–chrome alloy maxillary central incisor crown cemented on the Ti-base abutment. (**C**) Schematic representation of experimental set-up for step stress accelerated life testing.

Implants were embedded in polymethylmethacrylate acrylic resin (Jet; Classico Artigos Odontologicos, Sao Paulo, SP, Brazil) in a vertical position into a 15 mm diameter matrix using a dental surveyor (Delineador B2; Bio-Art, Sao Carlos, SP, Brazil). The implant platform was positioned at the level of the potting acrylic resin surface. After polymerization of the acrylic resin, proprietary Ti-base abutments (S.I.N. Implant System and Neodent) were tightened to the respective implants (Figure 2A). A digital torque gauge (Tohnichi BTG150CN-S, Tohnichi America, Buffalo Grove, IL, USA), was used to assess the final torque, as per the manufacturer’s recommendation (20 N·cm). 

Standardized cobalt–chrome alloy maxillary central incisor crowns were milled for each group and (Wirobond 280, BEGO, Lincoln, RI, USA) cemented on the Ti-base abutments using a self-adhesive dual-curing resin cement (Rely X U200, 3M Oral Care, St. Paul, MN, USA), as per manufacturer’s instructions (Figure 2B). 

### 2.2. Fatigue Testing

Three stress profiles for step stress accelerated-life testing (SSALT), based on the fatigue behavior of narrow implant systems previously published by our group [34], were used in the present study. Eighteen specimens per experimental group were distributed in a 3:2:1 ratio to mild (n = 9), moderate (n = 6) and aggressive (n = 3) stress profiles, named based on its step-wise load increase, as detailed elsewhere [24,29,31,33]. 

SSALT was performed using all-electric dynamic test equipment (ElectroPulsTM E3000 Linear-Torsion system, Instron, Norwood, MA, USA) with compression load applied thirty degrees off-axis (ISO 14801:2016) lingually at the incisal edge of the crown using a tungsten-carbide indenter as presented in Figure 2C. Samples were immersed in water, and testing was performed until failure (abutment, abutment screw, or implant fracture or bending) or survival (no failure at the end of the profile) at 15 Hz of frequency until a maximum load of 500 N. The number of cycles and the load at failure were recorded in function of the stress profiles, and the results were analyzed so that a profile of failure behavior could be extrapolated to normal conditions [31].

Data analysis consisted of an underlying life distribution as described elsewhere [31,35,36,37]. Weibull Distribution was chosen to fit the life data collected in SSALT. This model enabled the calculation and plot of the use level probability Weibull curves (Alta Pro 22; Reliasoft, Tucson, AZ, USA). 

The reliability was calculated for missions of 100,000 cycles at 100 and 150 N, relevant loads considering the physiological range. Statistical differences among groups were determined through the absence of overlap of the confidence intervals [31,36,37]. 

Failure analyses were performed in a polarized light stereomicroscope (AxioZoom V16, Zeiss, Oberkochen, Germany) using Z-stack mode that allowed for sequential imaging and extended depth of focus (ZEN 2.3 PRO, Zeiss), facilitating fractographic analysis. Subsequently, representative samples were evaluated via scanning electron microscopy (SEM, JEOL T220A, JEOL, Tokyo, Japan) to further assess the origin and direction of fracture propagation.

## 3. Results

**Figure 3:**
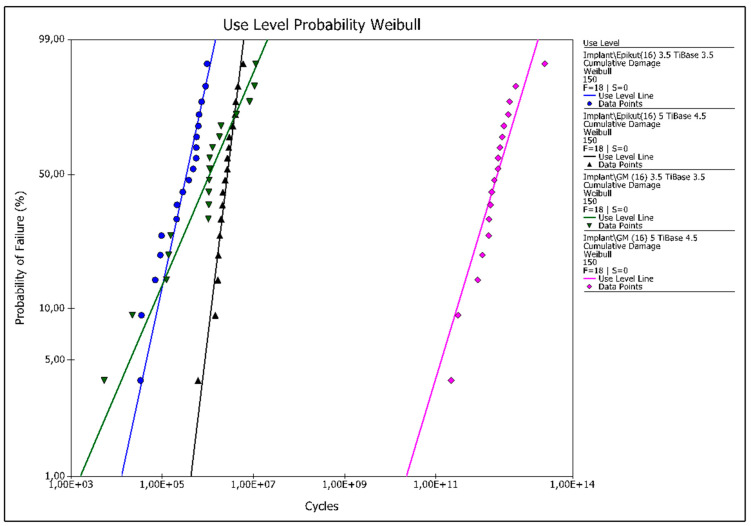
Use level probability Weibull curves of the experimental groups at a set load of 150 N.

All samples failed during fatigue testing. The resulting beta values for both narrow and wide Epikut groups were higher than 1 (1.28 and 2,29, respectively), suggesting that failures were dictated by fatigue damage accumulation. GM groups, otherwise, presented beta values lower than 1 for both narrow and wide groups (0.64 and 0.9, respectively), which suggests that failures in these groups were most likely dictated by material strength. The use level probability Weibull curves for a use level load of 100 N are presented in Figure 3.

### 3.1. Reliability 

The reliability of the experimental groups with their 90% CIs for missions of 100,000 cycles at 100 and 150 N is presented in Table 1. High reliability was noted for all implant systems investigated for missions at 100 N (>99%), regardless of the implant diameter.

For a mission of 100,000 cycles at 150 N, a slight reliability decrease was observed for narrow implant systems in comparison to its performance at 100 N. Wide-diameter implants presented significantly higher reliability than narrow implant systems. Furthermore, no significant differences between groups with the same implant diameter were observed regardless of manufacturer.

### 3.2. Failure Modes

**Figure 4:**
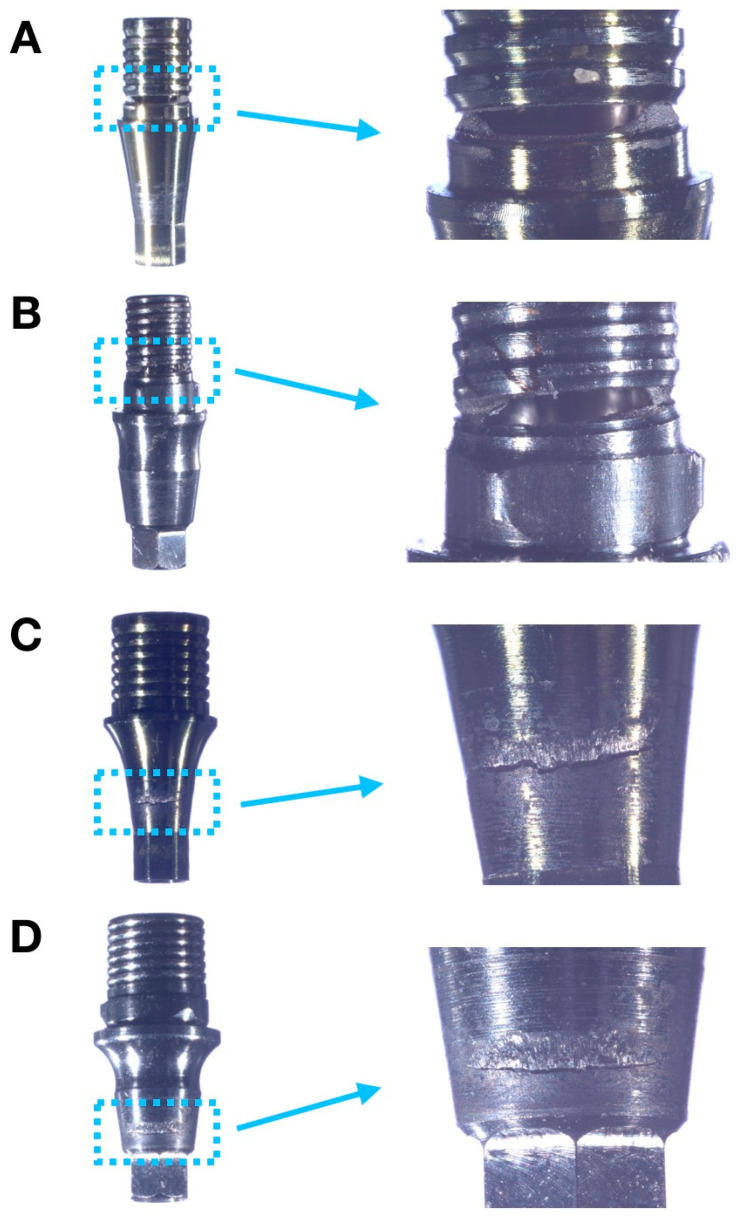
Failure modes of (**A**) Epikut 3.5, (**B**) GM 3.5, (**C**) Epikut 5, and (**D**) GM 5 experimental groups. Failures were comprised of Ti-base abutment fractures at the abutment platform for narrow systems (**A**,**B**) and at the area of connection with the implant for wide implant systems (**C**,**D**).

Representative failed specimens of each group are presented in Figure 4. Failure modes were chiefly comprised of Ti-base abutment fracture, mainly from lingual to buccal surface. No implant or screw fracture were observed for either narrow or wide implant systems.

**Figure 5:**
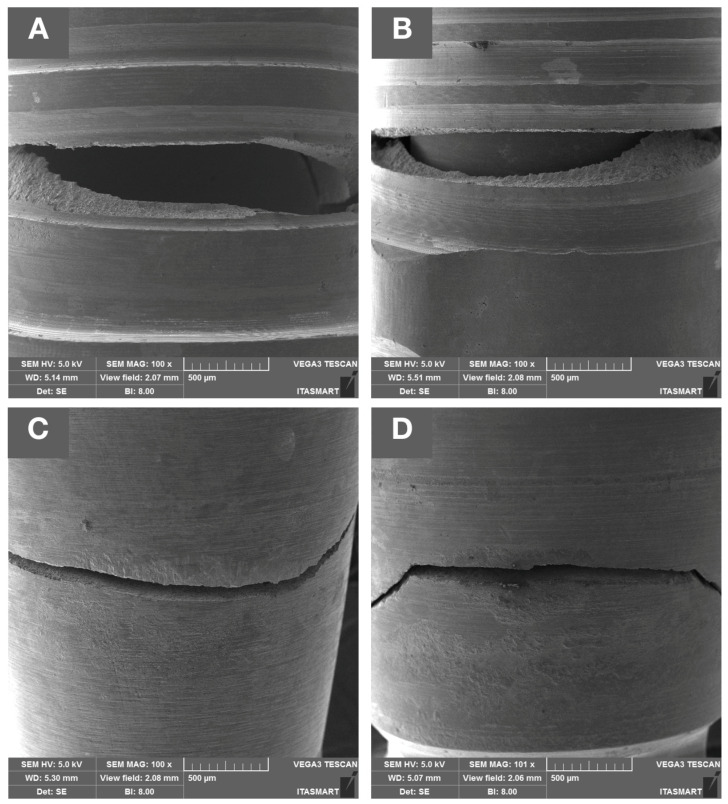
Scanning electron microscope images of representative samples of all experimental groups (**A**) Epikut 3.5, (**B**) GM 3.5, (**C**) Epikut 5, and (**D**) GM 5 failed during fatigue testing. While narrow implant systems presented Ti-base abutment fractures restricted to the abutment platform at the level where the crown is seated, Ti-base abutments of wide implant systems presented fractures mainly at the area of connection with the implant.

Failure analyses demonstrate similar fracture patterns within implants of the same diameter implant. While narrow implant systems presented Ti-base abutment fractures restricted to the abutment platform at the level where the crown is seated (Figure 4A,B), Ti-base abutments of wide implant systems presented fractures mainly at the area of connection with the implant (Figure 4C,D). Higher magnification images of the fractured areas obtained with SEM are depicted in Figure 5 for representative samples of all tested groups. 

## 4. Discussion

From a mechanical perspective, it has been suggested that dental implants with smaller bulk structure and their respective prosthetic components may be prone to fractures and mechanical complications [24,28,29]. The demand for dental implants with improved mechanical properties that allow for their indication in reduced diameters led to the development of cold-working processing and the development of titanium alloys with a proven superiority compared to commercially pure titanium in terms of mechanical properties [38,39]. The development of novel implant systems through different processing methods, bulk materials and designs, along with the well-known slow and irreversible crack propagation caused by the repeated physiological masticatory loading, demands an extensive evaluation of the mechanical properties of implant systems in conditions that closely simulate the cyclic loading present in the oral environment. In this effort, the current study sought to evaluate the fatigue performance and failure modes of Ti-base abutments supported by narrow dental implants and to compare them with wide-diameter dental implants. All systems tested presented high reliability for missions under physiological loads, whereas wide-diameter implant systems presented higher reliability at missions at 150 N in comparison to narrow implants. Therefore, the postulated null hypothesis that no significant differences would be observed in the reliability of narrow and wide implant systems regardless of the manufacturer was rejected.

The beta values obtained after fatigue testing suggest that failures were caused by fatigue damage accumulation for both Epikut groups and by material strength for GM groups. These findings are related to the combination of damage accumulation through repeated loading and to the strength of the weakest component of the assemblies, which in the tested scenario was the Ti-base abutment [31]. Fractographic analyses of the failed samples corroborated abutment fracture as the main failure mode after fatigue testing, with no fractures of any tested implant, regardless of its diameter or manufacturer. While implant fracture has been considered a rare complication that occurs in <1% of implants during a 5-year period [40], the more typical mode of single-implant retained crowns mechanical failure reported in dental literature is abutment screw loosening and fracture [41], with an 8.8% cumulative five-year complication rate [42]. The absence of abutment screw fracture in the present study may be explained by the significant improvements in the mechanical properties of Ti-6Al-4V alloy abutment screws, which may have eliminated the more typical mode of implant mechanical failure [43]. Furthermore, the modes of failure depicted in the present study varied as a function of implant/abutment diameter. While failures of the narrow systems were comprised of Ti-base abutment fractures at the abutment platform where the crown is seated, failures of wide-diameter implant systems were observed at the area of connection with the implant. As expected, failure occurred at the thinnest area of the respective Ti-base abutments, where the fatigue damage accumulation and material strength dictated the failure of the GM and Epikut groups, respectively. 

Reliability analyses at a given mission of 100 N for 100,000 cycles demonstrated a high probability of survival, almost 100%, for all tested implant systems. These results suggest that, irrespective of commercial system, narrow implants manufactured with grade IV commercially pure and processed using a cold-working method are a reliable option to rehabilitate the anterior area since mean physiologic masticatory forces in these regions vary within the range from 25 to 45 N [44]. At a given mission of 150 N, a 13% decrease in reliability was observed for both narrow implant systems in regard to their performance at 100 N. Such behavior is in agreement with the previous literature that investigated the fatigue behavior of narrow implant systems using step stress accelerated life-testing, where a decrease of approximately 10% was observed in missions at 150 N for narrow implant systems (3.5 mm) with internal conical connections compared to their performance at 100 N [24]. Moreover, as reported by the same study, the absence of implant body fractures after fatigue testing suggests that the increased contact of the abutment with the implant walls in internal conical connections could protect the implant integrity even when a challenging scenario of reduced wall thickness is present, as observed in the current study [24,29]. 

At given missions at 100 and 150 N for 100,000 cycles, wide-diameter implant systems tested in the present study exhibited high reliability, almost 100%, with no significant differences in their performance between missions at 100 and 150 N. Furthermore, at a given mission of 150 N, wide implant systems presented significantly higher reliability than narrow implant systems. Previous literature that investigated the physiological masticatory forces of human beings has suggested peak force variations from 100 to 140 N in molars and from 25 to 45 N in incisors [44]. Therefore, wide implant systems should be considered the first option to rehabilitate posterior edentulous areas whenever there is bone availability for their indication. Moreover, in silico literature has suggested that the implant diameter may have a significant influence on bone stress distribution, where wider-diameter implants may help to reduce bone stress [45].

The SSALT methodology and test configuration used in the current study have been frequently used to assess the mechanical behavior of dental implants, abutments and their respective superstructures, allowing for the extrapolation of clinical failure patterns in a timely way [23,29,30,31,32,33]. While results of the present study are encouraging, given that indication of these implants may benefit patients in avoiding bone grafting procedures, this is an in vitro study that represents preclinical screening and further clinical studies are warranted to characterize the multitude of variables regarding the treatment with narrow implants. 

## 5. Conclusions

Ti-base abutments supported by narrow and wide implant systems presented high reliability for physiologic masticatory forces. For high load-bearing applications, wide-diameter implants presented increased reliability. Failures were confined to abutment fractures.

## Figures and Tables

**Table 1:** Probability of survival (%) with 95% confidence intervals of the experimental groups for missions of 100,000 cycles at 100 and 150 N.

	Epikut 3.5 (3.5 Ti-Base)	GM 3.5 (3.5 Ti-Base)	Epikut 5 (4.5 Ti-Base)	GM 5 (4.5 Ti-Base)
Upper Bound	0.99	0.99	1	1
Probability of Survival 100 N	0.99 Aa	0.99 Aa	1 Aa	1 Aa
Lower Bound	0.98	0.94	0.99	1
Upper Bound	0.95	0.95	0.99	1
Probability of Survival 150 N	0.86 Bb	0.86 Ab	0.99 Aa	1 Aa
Lower Bound	0.61	0.62	0.99	0.99

Capital letters indicate significant differences between missions at 100 and 150 N, non-capital letters depict significant differences among experimental groups in the same mission.

## Data Availability

Data will be made available upon request.
